# Pneumocephalus and air travel: an experimental investigation on the effects of aircraft cabin pressure on intracranial pressure

**DOI:** 10.1038/s41598-020-70614-w

**Published:** 2020-08-12

**Authors:** Ee Lim, Boon Leong Lan, Ean Hin Ooi, Hu Liang Low

**Affiliations:** 1 School of Engineering, Monash University Malaysia, Jalan Lagoon Selatan, 47500 Bandar Sunway, Selangor Malaysia; 2 Advanced Engineering Platform, Monash University Malaysia, Jalan Lagoon Selatan, 47500 Bandar Sunway, Selangor Malaysia; 3 Department of Neurosurgery, Sunway Medical Centre, Jalan Lagoon Selatan, 47500 Bandar Sunway, Selangor Malaysia; 4 Department of Neurosurgery, Queen’s Hospital, Romford, London, RM7 0AG UK

**Keywords:** Biomedical engineering, Translational research

## Abstract

This study investigates the effects of aircraft cabin pressure on intracranial pressure (ICP) elevation of a pneumocephalus patient. We propose an experimental setup that simulates the intracranial hydrodynamics of a pneumocephalus patient during flight. It consists of an acrylic box (skull), air-filled balloon [intracranial air (ICA)], water-filled balloon (cerebrospinal fluid and blood) and agarose gel (brain). The cabin was replicated using a custom-made pressure chamber. The setup can measure the rise in ICP during depressurization to levels similar to that inside the cabin at cruising altitude. ΔICP, i.e. the difference between mean cruising ICP and initial ICP, was found to increase with ICA volume and ROC. However, ΔICP was independent of the initial ICP. The largest ΔICP was 5 mmHg; obtained when ICA volume and ROC were 20 ml and 1,600 ft/min, respectively. The postulated ICA expansion and the subsequent increase in ICP in pneumocephalus patients during flight were successfully quantified in a laboratory setting. Based on the quantitative and qualitative analyses of the results, an ICA volume of 20 ml and initial ICP of 15 mmHg were recommended as conservative thresholds that are required for safe air travel among pneumocephalus patients. This study provides laboratory data that may be used by doctors to advise post-neurosurgical patients if they can safely fly.

## Introduction

Craniotomy refers to the surgical removal of a portion of the skull to gain access to the contents of the intracranial cavity. The procedure usually involves exposing the brain after opening its membranous coverings^1^, which inevitably expose the human brain to surrounding air. Varying amounts of air within the intracranial cavity (ICA) (known as pneumocephalus) remain after craniotomy^2^. In most cases, pneumocephalus is not life-threatening as the ICA is normally absorbed into the systemic circulation over a period of several weeks^3–5^. Safety concerns arise when patients with pneumocephalus engage in air travel before the ICA is fully absorbed.

Air safety regulations require cabins of commercial aircraft to be pressurized to a standardized minimum of 75 kPa, which is approximately 75% that of atmospheric pressure at sea level (101.3 kPa), at cruising altitude^6,7^. Consequently, during air travel, the ambient pressure may be as much as 25 kPa lower than the initial pressure of the ICA, which is at 101.3 kPa. According to Boyle's law, the difference in pressure between the aircraft cabin and the ICA would result in an increase in the ICA volume, as the ICA expands to equilibrate with the cabin pressure. The expansion, which takes place inside the rigid cranium, may cause cerebral compression that can lead to elevations in the intracranial pressure (ICP). A mild increase in ICP is tolerable. However, if the increase in ICP is too large, cerebral herniation may occur, which severely endangers the life of the pneumocephalus patient.

In spite of the potential life-threatening event, pneumocephalus and air travel safety remains a controversial topic^8^. Cases of post-neurosurgical patients embarking on commercial air travel have been recorded in the past. However, the conclusion drawn on the severity of pneumocephalus during air travel varies among the different cases. For instance, Beda et al.^9^ reported on a parietotemporal craniectomy patient who developed deteriorating neurological condition and confirmed intraparenchymal pneumocephalus after embarking on air travel. Chue et al.^8^ reported on the development of extensive pneumocephalus in a facial trauma patient who engaged in air travel post-injury. However, there were no reported neurological symptoms. Sweni et al.^10^ reported on the case of a patient, who underwent cervical epidural injection, developing pneumocephalus after a 630 mile flight. The same authors also reviewed six cases of air travel-related pneumocephalus between 1991 and 2011. However, only one case was found to relate to the possible expansion of pre-flight pneumocephalus at cabin pressure. Wilson et al.^11^ reported no safety issues in a patient with pre-flight pneumocephalus who embarked on a 45 min commercial air travel, while Donovan et al.^12^ found no safety risks in the air evacuation of 21 wounded soldiers with pre-flight pneumocephalus.

The lack of clarity on air travel safety among post-operative neurosurgical patients is further demonstrated by the absence of consensus amongst neurosurgeons when addressing post-neurosurgery air travel. A survey conducted by Amato-Watkins et al.^13^ revealed that 5 out of 66 neurosurgeons were against air travel after neurosurgery, with the remaining 61 recommending an air travel restriction period ranging from 2 to 8 weeks^13^. The UK Civil Aviation Authority recommends that patients avoid flying in the first week after intracranial surgery^13^. A survey conducted by Seth et al.^2^ on 17 commercial airlines revealed that only three of them were able to provide detailed advice on whether post-craniotomy patients should embark on air travel.

There is presently no consensus amongst medical professionals and the airline industry on when patients can safely fly after cranial surgery, especially when pneumocephalus is present. Much of the advice used by the industry and the medical profession is not evidenced-based and relies on case reports, cranial CT scans in symptomatic patients following flights or mathematical models^14^. This is not surprising given the difficulties of performing in-flight brain imaging or ICP monitoring, which has prompted calls for more research into this topic^8,15^. There is currently insufficient information to show a direct correlation between the volume of intracranial air and changes in intracranial pressure during air travel. In this paper, we seek to answer the questions by developing a laboratory model that simulates a flight cabin, the human brain and skull. Through this model, we seek to demonstrate the effects of varying initial ICA volume, initial ICP and rate of change of external pressure on the ICP in the presence of pneumocephalus.

## Hydrodynamics of intracranial pressure

Intracranial pressure is defined as the pressure within the human skull and is commonly used as a measure of pressure in the brain and the cerebrospinal fluid (CSF). A healthy ICP ranges from 7 to 15 mmHg when the individual is in the supine position^16^. An ICP of 20 mmHg is considered to be abnormal and anything beyond 25 mmHg usually requires immediate medical attention^17^. Unfortunately, a general ICP threshold for cerebral herniation does not exist, although some studies have suggested that cerebral herniation may occur at ICP as low as 20 mmHg depending on the physiology of the patient^18^. ICP measurement can be performed invasively and non-invasively^19,20^. Invasive methods are more accurate, with ICP monitoring via catheters placed in the lateral ventricles being the ‘gold standard’ for measuring ICP.

**Figure 1:**
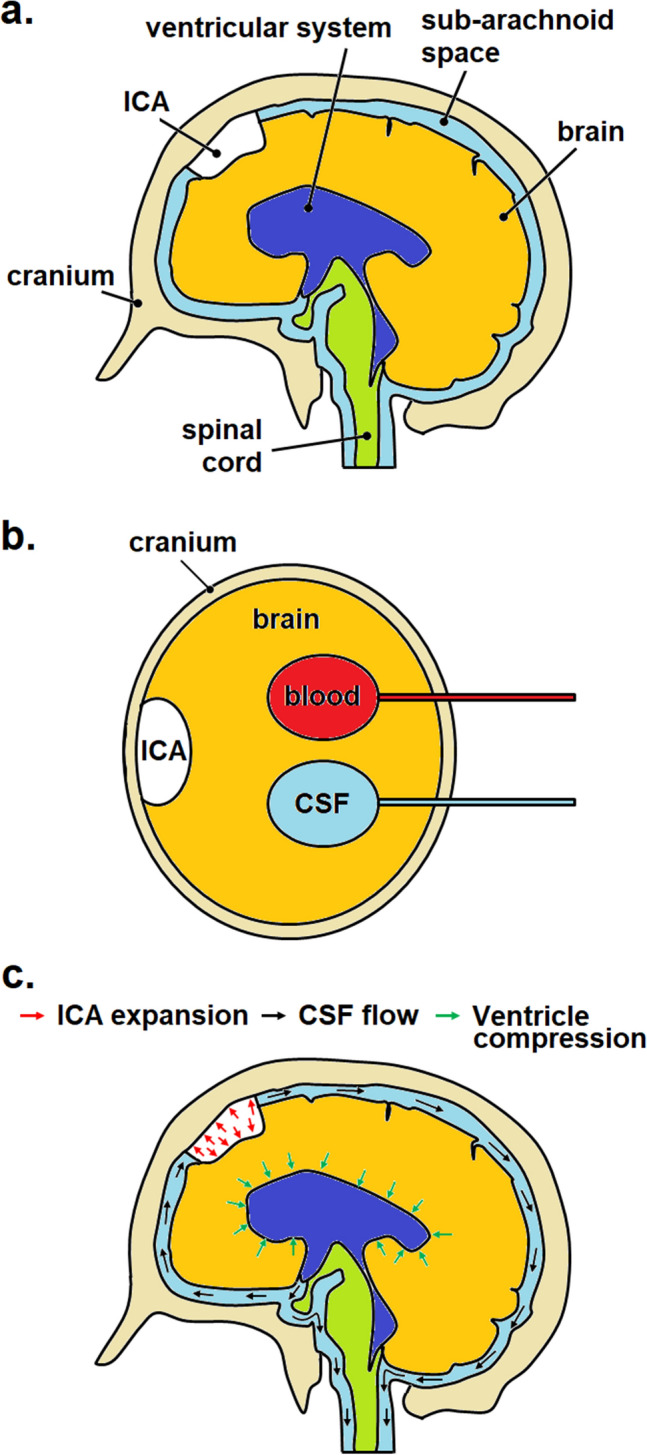
Hydrodynamics of intracranial pressure. (**a**) Schematic of intracranial system of a pneumocephalus patient, (**b**) model of the four compartments of the intracranial system in a pneumocephalus patient, (**c**) expansion of ICA (red arrows) that leads to ventricle compression (green arrows) and CSF flow (black arrows). CSF is contained in both the ventricular system and the sub-arachnoid space.

The hydrodynamics of ICP may be explained with the aid of a simplified schematic of the human head, which consists of the cranium, the ventricular system (lateral, third and fourth ventricles), the subarachnoid space, the brain tissue, the vascular system within the brain and in patient with pneumocephalus, the ICA. The schematic is illustrated in Fig. 1a. CSF is produced by the ventricular system, which flows from the lateral ventricles into the third ventricle and then the fourth ventricle. From here, the CSF flows into the subarachnoid space, which helps to cushion and protect the brain from the rigid cranium.

Hydrodynamically, the intracranial system of a healthy individual can be simplified into three components, i.e. brain, CSF and blood^21^. In a pneumocephalus patient, a fourth component, the ICA, is present. This is shown in Fig. 1b. According to the Monro-Kellie doctrine, the sum of volume from each component must remain constant. In other words, an increase in volume in any one of the components will result in an equivalent decrease in volume in the others. In the case of a pneumocephalus patient embarking on air travel, the expansion of ICA in response to the smaller cabin pressure will lead to a decrease in the volume of the brain, the CSF and blood, which subsequently raises the ICP. The process generally involves the displacement of CSF and blood from the cranium in order to compensate for the increase in ICA volume, such as shown in Fig. 1c.

## Materials and methods

### Experimental setup

#### The pressure chamber

**Figure 2:**
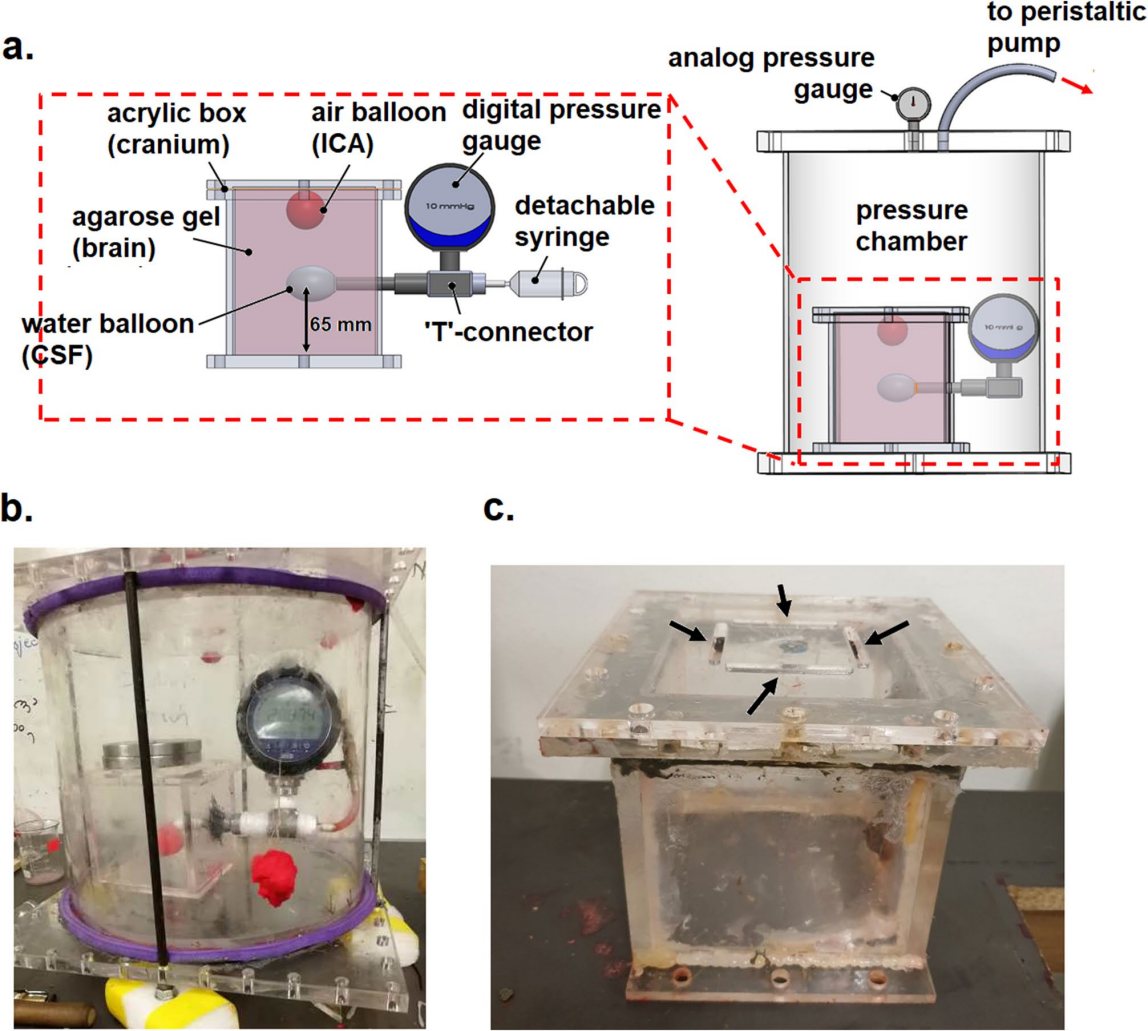
The experimental setup employed in the present study. (**a**) Schematic of the setup depicting the air balloon, water balloon and digital pressure gauge. (**b**) Actual experimental setup. (**c**) Model of the human cranium (black arrows indicate the grooves cut into the top lid).

A custom-made pressure chamber (see Fig. 2a) was constructed to replicate the aircraft cabin. The pressure chamber was constructed from acrylic of thickness 5 mm, with diameter and height of 350 and 300 mm, respectively. The top lid of the pressure chamber was connected to a peristaltic pump (CATALYST FH100, Cole-Palmer) that was used to depressurize the chamber from atmospheric pressure (101.3 kPa) to cabin pressure at cruising altitude (75 kPa). An analog pressure gauge was installed to monitor the pressure inside the chamber.

#### The cranium model

The cranium was modelled using an acrylic box measuring 120 mm in width, height and depth. The dimensions were chosen so that the box has sufficient capacity to contain the brain model (see “The brain model” section), the ICA model (see “The ICA model” section) and the CSF model (see “The CSF model” section).

#### The brain model

To avoid complications in handling actual brain tissues, 0.4% agarose gel (1st BASE, BIO-1000) was used as a substitute (see Fig. 2a). In particular, the 0.4% concentration was chosen as it has been shown to exhibit mechanical properties that are similar to that of human brains^22^. The volume of human brain can range from 1,052.9 to 1,498.5 cm^3^ among men and from 974.9 to 1,398.1 cm^3^ among women^23^. The volume of agarose gel employed in the present study varies depending on the volume of the ICA model (see “The ICA model” section), but falls within the range of approximately 1662 to 1702 cm^3^. This is acknowledged to be slightly larger than the range reported in the literature but was deemed to be acceptable for the purpose of the present study.

#### The ICA model

Post-craniotomy ICA are commonly found in the subdural space over the frontal lobe or lobes^24^. In order to control the volume of ICA and its position within the cranium model, an air-filled latex balloon (see Fig. 2a) was used to model the ICA. The volume of the inflated balloon represents the volume of ICA. Since the shape of the cranium was not taken into consideration in the present study (see “The cranium model” section), it was assumed that the frontal lobe coincided with the lid of the cranium model. Hence, the air balloon was positioned at the centre and just beneath the lid of the cranium model. Four grooves were cut into the lid of the acrylic box, such as shown in Fig. 2c, in order to allow the ICA to react with the changes in air pressure inside the pressure chamber.

#### The CSF model

The component representing the CSF in the experimental setup plays a vital role during the experiments, as it is also used to obtain the ICP. The CSF model was constructed using a water-filled balloon that was fitted to the cranium model approximately 65 mm from the base (Fig. 2a). The subarachnoid space between the brain and the cranium was not considered in the present setup; hence, all the CSF was assumed to be contained inside the ventricular system, which is modelled by the aforementioned water balloon. The balloon was filled with 16 ml of water, which approximated the volume of CSF inside the lateral ventricles of a normal middle-aged male adult^25^.

Outside of the cranium model, the water balloon was connected to a ‘T’-shaped joint, where one end was connected to a highly sensitive digital pressure gauge (WIKA CPG1500) with a measuring range of 75 mmHg and a precision of ± 0.015 mmHg, while the other end was connected to a syringe via a valve (Fig. 2a). The ICP was represented by the hydrodynamic pressure of the water balloon, which was measured using the digital pressure gauge. Different initial values of ICP were replicated by pressurizing the water balloon using the syringe. The valve allowed the syringe to be detached from the ‘T’-joint without affecting the pressure inside the water balloon.

#### Hydrodynamics of the experimental setup

The setup was designed specifically to replicate the hydrodynamics of ICP within the intracranial system. As the pressure chamber is depressurized to 75 kPa, the air balloon expands according to Boyle’s law. Since all compartments are contained inside the rigid skull, the expansion of air balloon compresses the agarose gel and subsequently the water balloon. Compression of the water balloon causes water to displace out of the skull, which is then recorded as a rise in ICP using the digital pressure gauge. It was not possible to measure the pressure of the brain model with the current setup. This is because the gel was used solely to transmit the force due to the expansion of ICA to the CSF model.

### Experimental procedure

The experiment begins with the setup of the CSF model, where the water balloon was pressurized using the syringe until the desired pressure, which was read from the digital pressure gauge, was reached. The pressure of the water balloon represented the initial ICP.

Agarose gel at 0.4% concentration was then prepared by mixing 7.6 g of agarose gel powder into 1.9 L of water. The mixture was brought to a boil and then placed in a water bath at room temperature for cooling under constant stirring. Once the temperature has dropped to approximately 40 °C, the mixture was poured into the cranium model. The air balloon that has been inflated to a pre-determined volume was then placed onto the liquid agarose gel mixture and the lid of the box was carefully secured, displacing some agarose gel mixture in the process. Care was taken to ensure that the air-filled balloon remained positioned at the centre of the box and that no air pockets were trapped between the gel and the lid. The mixture was then allowed to set for a minimum of 18 h with the air balloon in place.

The setting process was found to cause slight deviations in the initial ICP from the intended value. As the effect was unavoidable and efforts to compensate for the deviation by pressurizing or depressurizing the water balloon after the gel has set led to unfavourable outcome, it was decided that deviation in the initial ICP that was greater than 25% was unacceptable, in which case, the setup was discarded.

Once the gel has set, the syringe was detached from the water balloon and the rest of the setup was carefully transferred into the pressure chamber, where it was depressurized to 75 kPa at a fixed flowrate using the peristaltic pump. The depressurization represents the ascension of the aircraft from sea level to cruising altitude. At 75 kPa, the pump was stopped and the pressure inside the chamber was held constant for 5 min. This represented the cruising phase of the aircraft. After 5 min, the vacuum chamber was pressurized back to sea level at the same flowrate as depressurization and held at that pressure for a further 90 s before the experiment was terminated. During the entire experiment, readings from the digital pressure gauge were recorded at 5 min interval.

Each experiment was carried out three times, where the recorded ICP values were averaged prior to further analysis.

### Experimental parameters

The effects of three different parameters on the expansion of ICA and the rise in ICP were investigated in the present study. These parameters are the ICA volume, the rate of climb (ROC) of the aircraft and the initial ICP.

ICA volumes reported among pneumocephalus patients vary depending on various factors and circumstances. A medical report by Donovan et al.^12^ presented a case study of 21 pneumocephalus patients with ICA volumes ranging from 0.6 to 42.7 ml, indicating its huge variability. Accordingly, five values of ICA volume that cover the aforementioned range of ICA volumes were investigated, i.e. 10, 15, 20, 30, 40 and 50 ml.

The aircraft ROC is defined as the rate of ascension of the aircraft from sea level to cruising altitude and is usually expressed in ft/min. ROC influences the rate of change of the cabin pressure, which in the present study, is represented by the rate of change of the pressure inside the pressure chamber from 101.3 kPa (sea level) to 75 kPa (cruising altitude). The ROC of commercial aircrafts during a typical flight varies depending on factors such as weight, type and speed of the aircraft. According to the Aeronautical Information Manual (https://www.faa.gov/atpubs), the climb should be performed at an optimum rate consistent with the operating characteristics of the aircraft from sea level to 1,000 ft, after which the ROC should be between 500 and 1,500 ft/min until the assigned altitude is reached. In order to simplify and to relate the rate of change of pressure inside the pressure chamber to the ROC, the latter was assumed to be constant so that the rate of depressurization of the pressure chamber from 101.3 to 75 kPa was also constant. ROC values of 960 and 1,600 ft/min were investigated in the present study as they approximately represent the median and the upper limits of the operational ROC of commercial aircrafts. ROC of 960 and 1,600 ft/min were achieved by operating the peristaltic pump at operating speeds of 250 and 400 rpm, respectively.

Normal ICP in adults can range from 5 to 15 mmHg, depending on factors such as age and pre-existing medical conditions^18,26–28^. Hence, five initial ICP values, given by 5, 10, 15, 20 and 25 mmHg, were investigated in the present study. Values of 20 and 25 mmHg were selected to replicate conditions of mild intracranial hypertension.

## Results

### Transient ICP response

**Figure 3:**
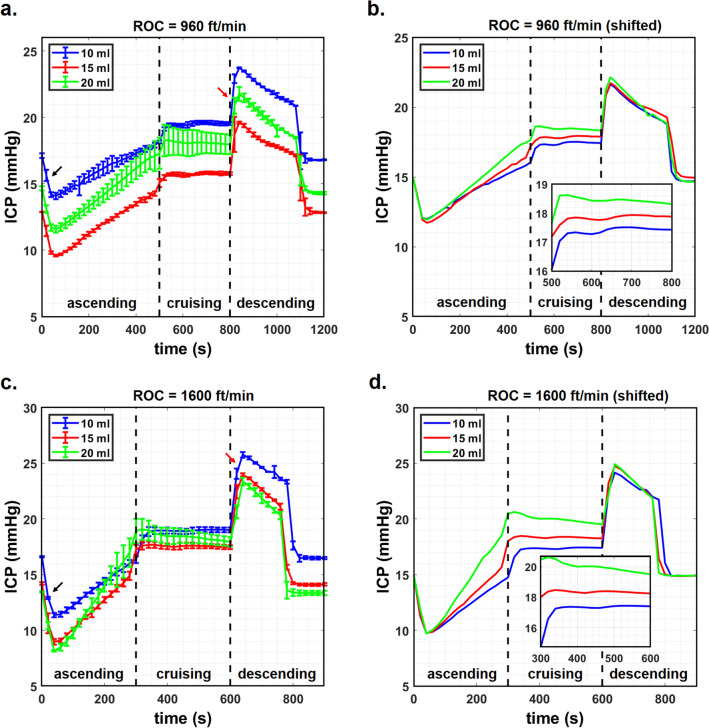
Transient ICP response at ICA volume of 10, 15 and 20 ml. (**a**) ROC = 960 ft/min, (**b**) ROC = 960 ft/min after data oset, (**c**) ROC = 1,600 ft/min, (**d**) ROC = 1,600 ft/min after data offset for a targeted initial ICP of 15 mmHg. Inset: Enlarged view of the ICP response during cruising stage.

Figure 3a, c plot the transient ICP responses obtained at ROC of 960 and 1,600 ft/min, respectively for ICA volumes of 10, 15 and 20 ml and an initial ICP targeted at 15 mmHg. As pointed out in “Experimental procedure” section, the process of setting the agarose gel around the CSF model led to slight variations in the actual initial ICP readings. Values of the actual initial ICP are summarized in Table 1, which are also reflected by the different starting ICP points in Fig. 3a, c.

**Table 1:** Summary of ICP responses at a targeted initial ICP of 15 mmHg.

Response (mmHg)	ICA volume (ml)		
	10	15	20
**ROC = 960 ft/min**			
Actual initial ICP	17.10 ± 0.18	12.88 ± 0.02	14.64 ± 0.16
Mean ICP at cruising	19.40 ± 0.36	15.65 ± 0.19	18.04 ± 0.22
ΔICP	2.30 ± 0.54	2.77 ± 0.21	3.40 ± 0.38
**ROC = 1,600 ft/min**			
Actual initial ICP	16.60 ± 0.05	14.22 ± 0.12	13.45 ± 0.05
Mean ICP at cruising	18.75 ± 0.68	17.55 ± 0.10	18.45 ± 0.33
ΔICP	2.15 ± 0.73	3.33 ± 0.22	5.00 ± 0.38

ΔICP = mean ICP at cruising – actual initial ICP.

The plots in Fig. 3a, c may be divided into three stages that corresponded to the three stages of flight, i.e. the ascending, cruising and descending stages. The start of the ascending stage was accompanied by a drop in ICP (see black arrows) for approximately 60 s. This was followed by a steady rise in ICP until the cruising stage, where the ICP throughout remained almost constant. The start of the descending stage saw a spike in ICP (see red arrows) that was followed swiftly by a decrease until the ICP returned to its initial value, after which it remained steady until the end of the experiment.

The drop and the rise in ICP immediately following the ascending and descending stages were not expected of the ICP response, as one would assume the ICP to only increase during the ascending stage and to only decrease during the descending stage. Upon further investigation, these anomalies were found to be experimental artefacts due to the partial exposure of the gel surface (see “The ICA model” section) to the pressure changes inside chamber and the use of the highly sensitive digital pressure gauge for recording the ICP. This will be discussed further in “Discussions” section. On the other hand, the observation that ICP during the cruising stage remained almost constant suggests that the aforementioned experimental artefacts have very little influence over the ICP reading when pressure inside the chamber is held steady. This allowed proper and reliable analysis to be carried out on the data during the cruising stage, which is of primary interest in the present study, as the ICP readings during the cruising stage corresponded to the stage when the cabin pressure is at its lowest.

### Effects of ICA volume ≤ 20 ml and ROC

The plots in Fig. 3a, c do not depict clearly the influence of ICA volume and ROC on ICP due to the slight variation in the actual initial ICP. For better visualization, the data used for plotting Fig. 3a, c were offset so that the initial ICP coincided with the target initial ICP of 15 mmHg. These revised plots are shown in Fig. 3b, d for ROC of 960 and 1,600 ft/min, respectively. A larger ICA volume led to a more rapid increase in ICP during the ascending stage, as indicated by the different slopes. Similarly, larger ICA volumes led to larger overall ICP during the cruising stage. The ICA volume did not appear to have any significant effect on the rate of ICP decrease during the descending stage.

**Figure 4:**
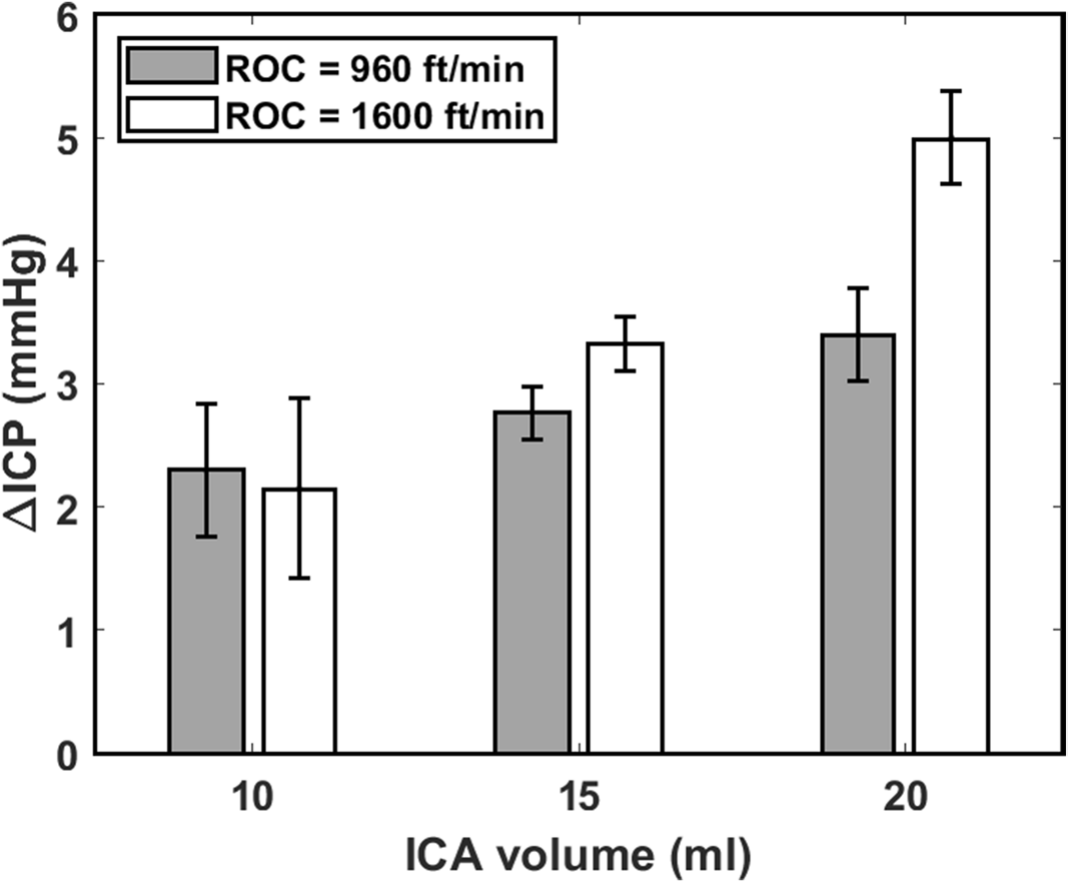
Plot of ∆ICP against ICA volume at ROC of 960 and 1,600 ft/min and targeted initial ICP of 15 mmHg.

Values of the ICP averaged over the 5 min cruising stage (ICP_cruise_) and the difference between ICP_cruise_ and the actual initial ICP (ΔICP) are summarized in Table 1. At ROC of 960 ft/min, an increase in ICA volume from 10 to 15 ml and from 15 to 20 ml led to 20.4 and 22.7% increase in ΔICP, respectively. Corresponding values obtained at ROC of 1,600 ft/min were 54.8 and 50.2%. Figure 4 plots the values of ΔICP against ICA volume obtained for ROC of 960 and 1,600 ft/min. Except for the case when ICA volume was 10 ml, a higher ROC resulted in a larger ΔICP. Nevertheless, the overlapping error bars observed for ICA volume of 10 ml could suggest that the difference in ΔICP obtained for ROC of 960 and 1,600 ft/min is inconclusive. At 15 and 20 ml, the values of ΔICP obtained at ROC = 1,600 ft/min were 16.8 and 32% larger than those obtained at ROC = 960 ft/min. The observations that ΔICP increased with ICA volume and ROC agree with the theoretical predictions made using the mathematical model of Andersson et al.^14^.

The plots illustrated in Fig. 3a, c along with the data presented in Table 1 showed that ICP_cruise_ did not exceed the 20 mmHg threshold of what is considered to be abnormal ICP. This may be due to the small variation in the actual initial ICP that may have influenced ICP_cruise_. For instance, a value of ΔICP = 5 mmHg was obtained at ROC = 1,600 ft/min and ICA volume of 20 ml. However, the value of ICP_cruise_ obtained was only 18.45 mmHg since the actual initial ICP was 13.45 mmHg. If ΔICP is independent of initial ICP, then the cruising ICP would have exceeded the 20 mmHg threshold in the event the initial ICP was 15 mmHg or greater.

### Effects of ICA volume ≥ 30 ml

**Figure 5:**
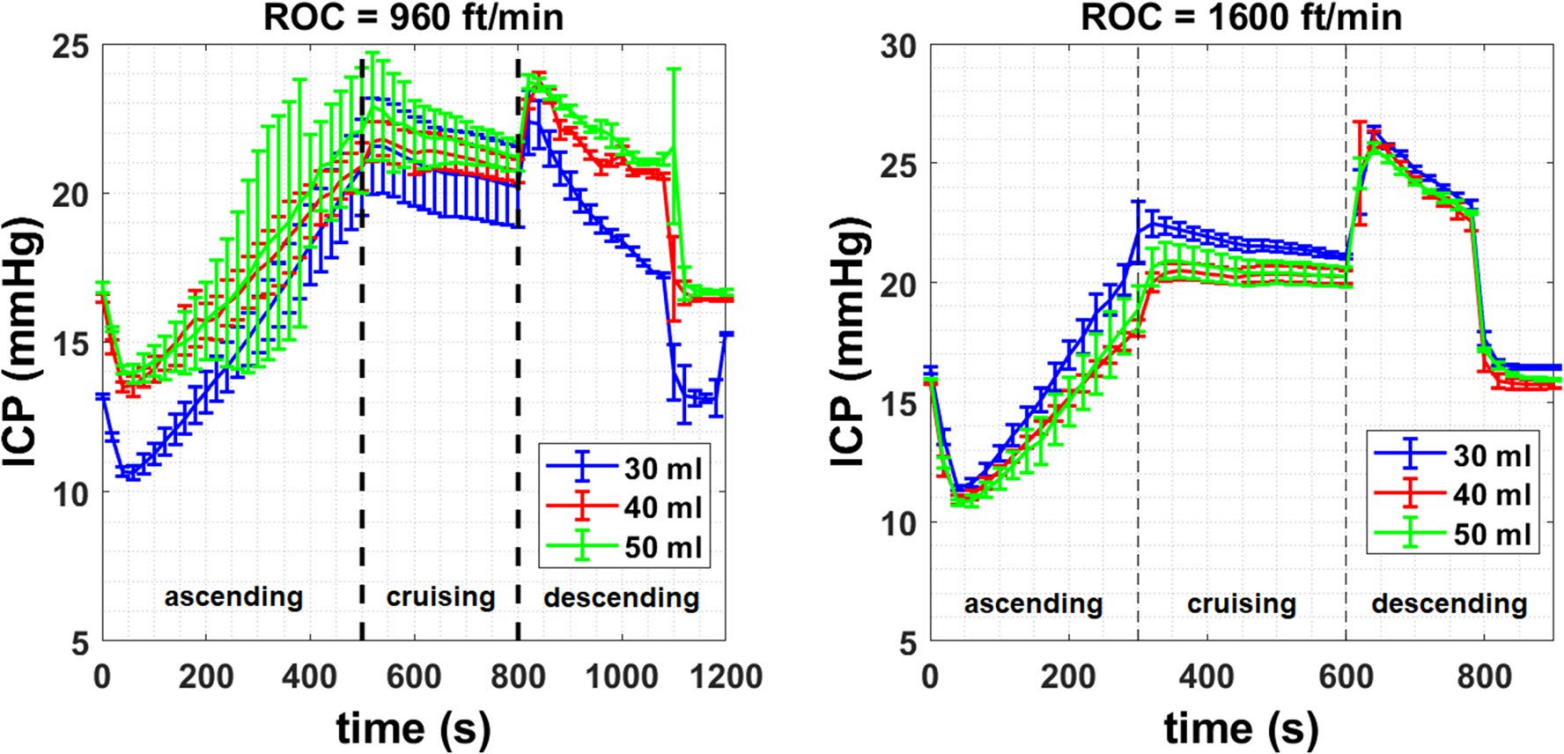
Transient ICP responses obtained for ICA volumes of 30, 40 and 50 ml, targeted initial ICP of 15 mmHg and ROC of **a** 960 and **b** 1,600 ft/min.

The results presented in “Transient ICP response” and “Effects of ICA volume ≤ 20 ml and ROC” sections have not included the data obtained for the cases when the ICA volumes were set to 30, 40 and 50 ml. This is due to the inconsistent responses observed among individual experiments from these cases. Figure 5 plots the transient ICP responses obtained at ROC of 960 and 1,600 ft/min, respectively for ICA volumes of 30, 40 and 50 ml and a targeted initial ICP of 15 mmHg. The trends observed were similar to those seen in Fig. 3a, c. However, the standard errors (particularly in the case of ROC = 960 ft/min) were larger and the overlaps among the different ICA volume indicated huge variability and poor reliability of the data collected. Moreover, the ICP showed a progressive decline during the 5 min cruising stage, unlike in Fig. 3a, c, where the ICP was almost constant.

**Figure 6:**
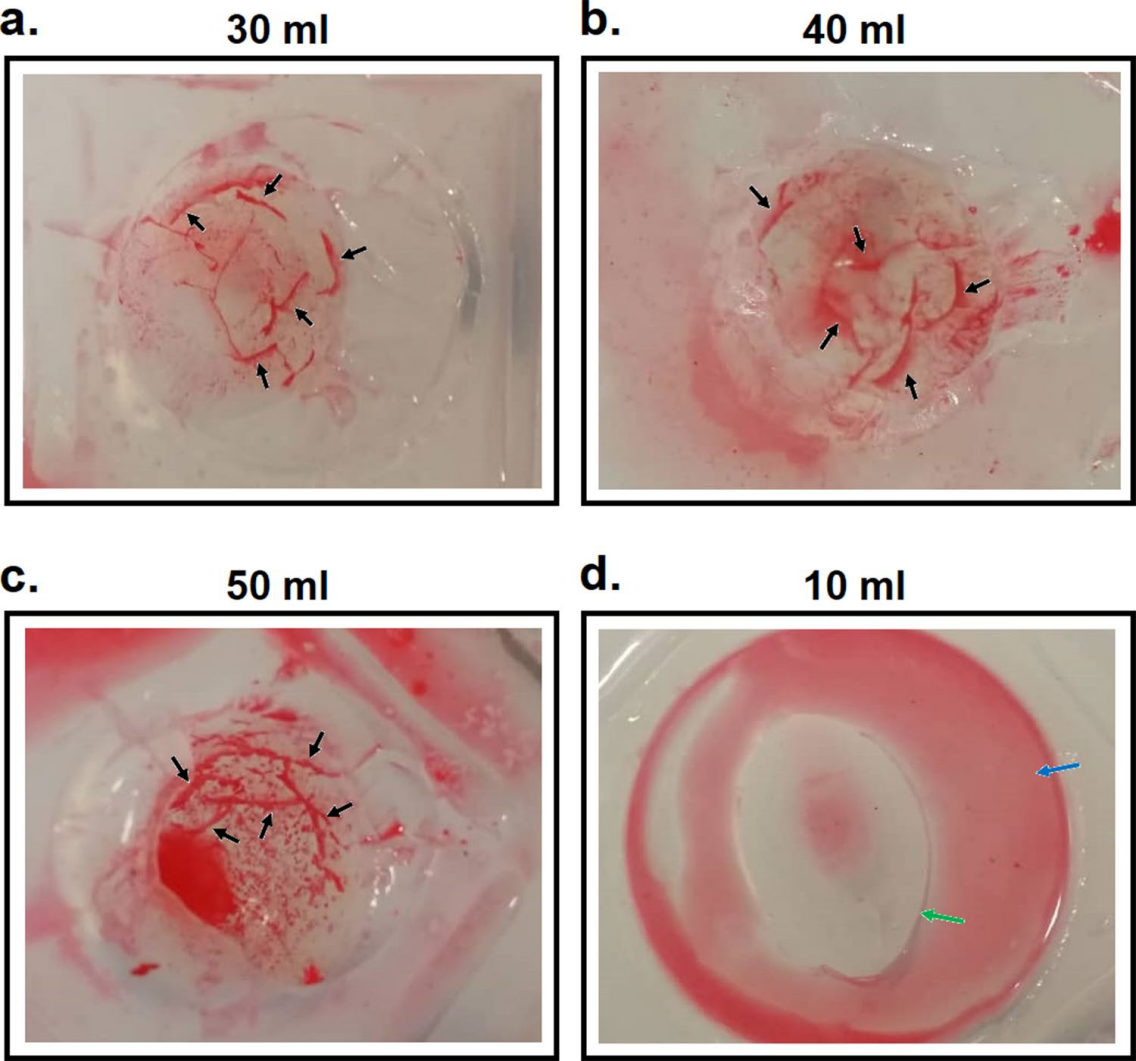
Evidences of cracks (black arrows) on the surface of the agarose gel following expansion of ICA volumes of (**a**) 30, (**b**) 40 and (**c**) 50 ml. Absence of cracks for ICA volume of 10 ml shown in panel (**d**). Blue arrow indicates pool of red water collected on the surface of gel, while green arrow points to the crater formed by the air balloon.

Upon further examination, it was found that cracks formed across the surfaces of the agarose gel where the air balloon was situated. This is illustrated in Fig. 6a–c, where the cracks can be identified by the red strips (see black arrows) that were present after dyed water was swiped across the surface of the gel post experiment. In contrast, the absence of these cracks was apparent in the experiments performed for ICA volume of 10, 15 and 20 ml (see Fig. 6d for the 10 ml case). The formation of these cracks suggests that the expansion of the air balloon was so large that the stress on the agarose gel was sufficiently high to cause it to crack. The presence of cracks increases the total surface area that is in contact with the air balloon. Under constant expansion force, this leads to a drop in the stress experienced by the gel (stress = force/area) and less compression on the water balloon. This may explain the gradual drop in ICP_cruise_, which is likely caused by the continuous propagation of cracks. The process of crack formation and propagation within the gel may have contributed to the large standard errors of the ICP reading seen in Fig. 5.

Table 2 summarizes the ICP responses obtained from the experiments performed for ICA volumes ≥ 30 ml. Mean values of the peak ICP obtained during the cruising stage (ICP_peak_) are also presented. Values of ΔICP obtained at ICA volumes of 30, 40 and 50 ml were at least two times larger than those found for ICA volumes of 10, 15 and 20 ml for the same ROC. However, the trend where ΔICP increases with ICA volume was not observed for ICA volumes of 30, 40 and 50 ml. Values of ΔICP at ROC of 960 ft/min were found to be larger than those at ROC of 1,600 ft/min, which was opposite to what was found for the cases when ICA volumes were smaller than 30 ml. It is important to note on the uncertainties that arose from the formation of cracks in the agarose gel during the experiments, which may have influenced the pressure readings and subsequently the interpretation of the data in Table 2.

**Table 2:** Summary of the ICP responses at a targeted initial ICP of 15 mmHg and ICA volumes of 30, 40 and 50 ml.

Response (mmHg)	ICA volume (ml)		
	10	15	20
**ROC = 960 ft/min**			
Actual initial ICP	13.21 ± 0.07	16.39 ± 0.15	16.76 ± 0.16
ICP_cruise_	19.47 ± 0.44	21.25 ± 0.35	22.89 ± 0.91
Mean peak ICP at cruising	20.15 ± 1.58	21.87 ± 0.58	24.47 ± 1.82
ΔICP	6.26 ± 0.51	4.86 ± 0.50	6.13 ± 1.07
**ROC = 1,600 ft/min**			
Actual initial ICP	16.49 ± 0.15	15.78 ± 0.03	15.97 ± 0.03
ICP_cruise_	21.39 ± 0.37	20.22 ± 0.57	20.96 ± 0.45
Mean peak ICP at cruising	21.99 ± 1.29	20.54 ± 0.35	21.61 ± 0.95
ΔICP	4.90 ± 0.52	4.44 ± 0.60	4.99 ± 0.48

### Effects of initial ICP

The effects of initial ICP on the transient ICP response were investigated for initial ICP values set to 5, 10, 15, 20 and 25 mmHg. The ROC was set to the median value of 960 ft/min. The ICA volume in this case was fixed at 20 ml, as this volume has been determined to lead to the largest ΔICP (see “Effects of ICA volume ≤ 20 ml and ROC” section). Consequently, the results obtained in this section would represent the worst-case-scenario for ΔICP. Conversely, ICA volume of 30 ml or greater was not considered due to the uncertainties arising from the formation of cracks (see “Effects of ICA volume ≥ 30 ml” section).

**Figure 7:**
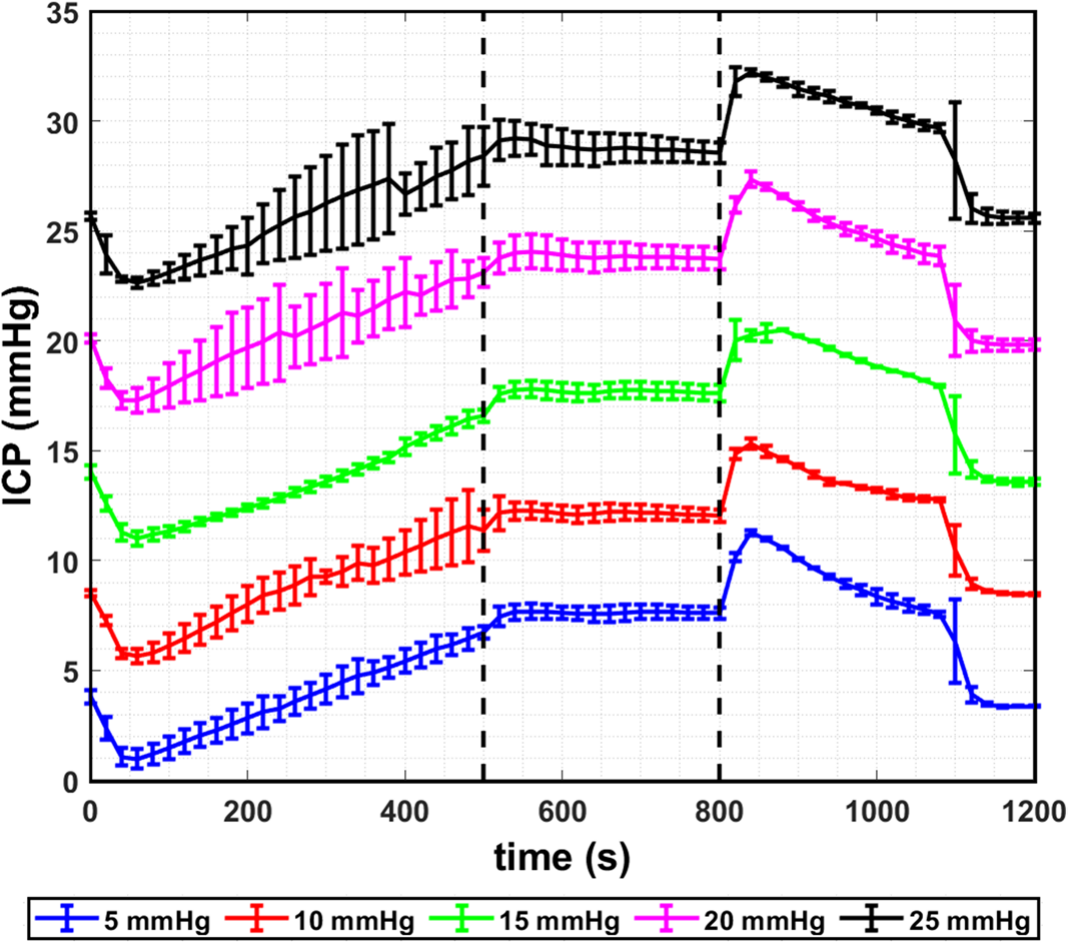
Transient ICP responses for targeted initial ICP of 5, 10, 15, 20 and 25 mmHg obtained for ICA volume of 20 ml and ROC of 960 ft/min.

**Figure 8:**
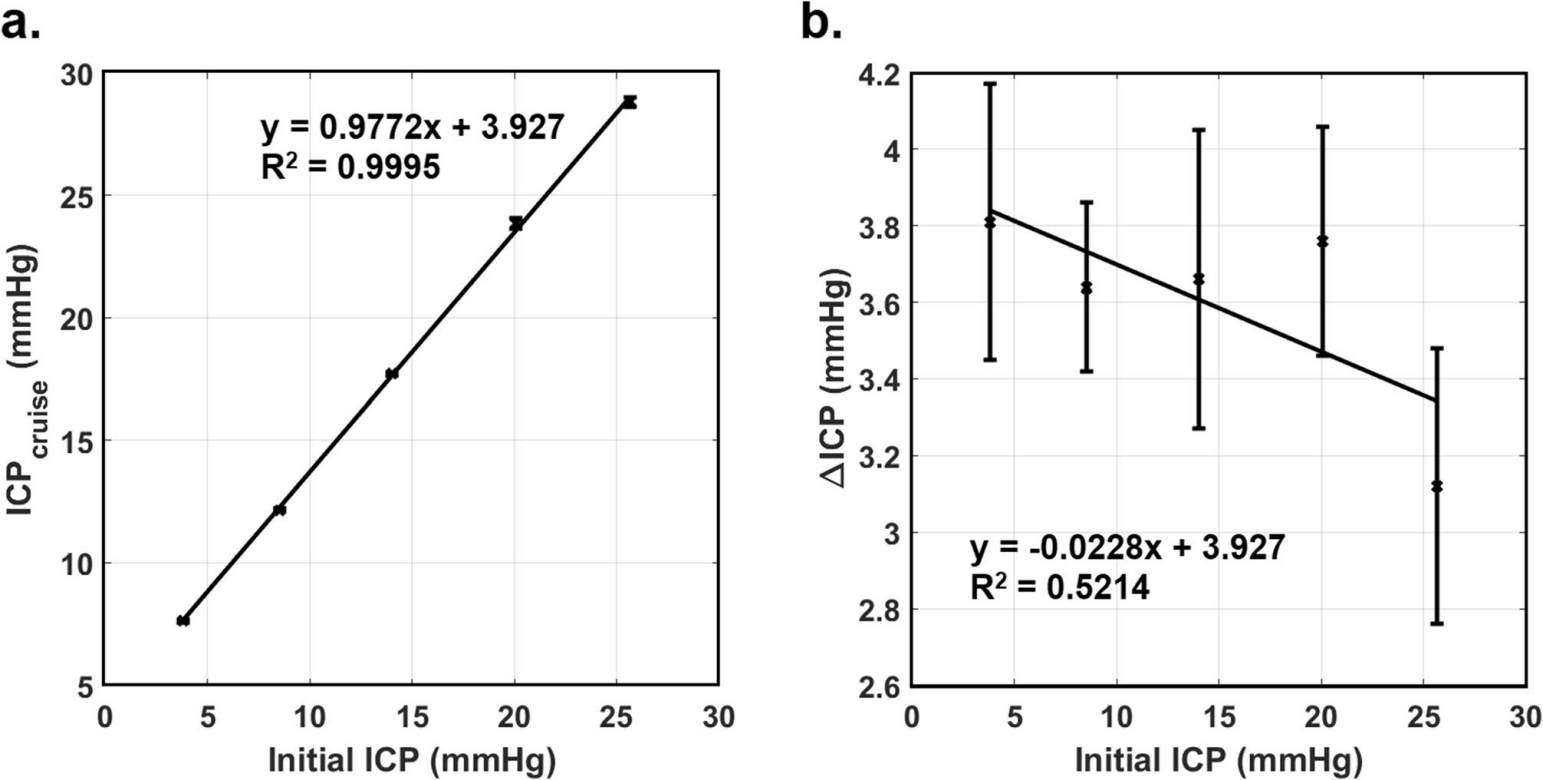
Plots of (**a**) ICP_cruise_ against the actual initial ICP and (**b**) ΔICP against the actual initial ICP for ICA volume of 20 ml and ROC of 960 ft/min.

Figure 7 plots the transient ICP responses obtained, while a summary of the ICP responses is presented in Table 3. As in the earlier cases, the actual initial ICP deviated slightly from the targeted initial ICP due to the setting of agarose gel. It was found that a larger initial ICP led to larger values of ICP_cruise_. However, values of ΔICP did not appear to be influenced by initial ICP. From Table 3, one may observe that the values of ICP_cruise_ did not exceed the abnormal threshold of 20 mmHg in cases where the initial ICP was 15 mmHg and smaller. On the other hand, initial ICP of 20 and 25 mmHg resulted in abnormal ICP_cruise_ values. Variation of ICP_cruise_ and ΔICP against the actual initial ICP are shown in Fig. 8a, b, respectively, where first order polynomials were fitted to the data of ICP_cruise_ and ΔICP. There was strong and significant correlation between ICP_cruise_ and initial ICP (*R*^2^ = 0.9995, *p* = 4.7 × 10^−6^), while the correlation between ∆ICP and initial ICP was moderate but not statistically significant (*R*^2^ = 0.5214, *p* = 0.17). Using the equation of linear fit shown in Fig. 8a, it is possible to estimate the critical value of initial ICP that will result in ICP_cruise_ that exceeds the 20 mmHg threshold of what is considered to be abnormal ICP. Hence, for a pneumocephalus patient with 20 ml of ICA who is travelling in an aircraft with ROC of 960 ft/min, an initial ICP of 16.45 mmHg or more would have resulted in ICP_cruise_ to approach levels that are considered to be abnormal. On the other hand, an initial ICP greater than 21.56 mmHg, which has already breached the unhealthy threshold, may lead to conditions that require immediate medical assistance (ICP > 25 mmHg).

**Table 3:** Summary of the ICP responses for different initial ICP at ICA volume of 20 ml and ROC = 960 ft/min.

Targeted initial ICP (mmHg)	Actual initial ICP (mmHg)	Mean ICP at cruising (mmHg)	ΔICP (mmHg)
5	3.81 ± 0.30	7.62 ± 0.06	3.81 ± 0.36
10	8.52 ± 0.15	12.16 ± 0.07	3.64 ± 0.22
15	14.04 ± 0.32	17.70 ± 0.07	3.66 ± 0.39
20	20.06 ± 0.20	23.85 ± 0.10	3.76 ± 0.30
25	25.68 ± 0.16	28.88 ± 0.20	3.12 ± 0.36

## Discussions

An experimental setup that investigates the expansion of ICA and its role in ICP elevation in a pneumocephalus patient during air travel was proposed in the present study. The setup utilizes a custom-made pressure chamber to recreate the reduced pressure environment inside the cabin of a flying aircraft, while the hydrodynamics of ICP was replicated using agarose gel for the brain, water for the CSF and balloon for the ventricles. Using the proposed experimental setup, we demonstrated for the first time the effects of air travel on pneumocephalus patients, which prior to this, were postulated based on Boyle’s law and the Monro-Kellie doctrine. In particular, observations made during the experiments supported the theory that the reduced pressure of aircraft cabins during flight can lead to expansion of ICA and the subsequent elevation in ICP.

Results from the experimental study indicated that the increase in ICP during cruising depended on the ICA volume and the ROC, but not significantly on the initial ICP. In particular, a larger ICA and a higher ROC resulted in higher elevations in the ICP. These behaviors complemented the predictions made by Andersson et al.^14^ using a hydrodynamics model of the intracranial system that also incorporated the presence of ICA. There was however, poor quantitative match between the experimental findings from the present study and the mathematical predictions of Andersson et al.^14^. For instance, the present study obtained an increase in ICP in the range of 2.3–3.14 mmHg at ROC of 960 ft/min, while the model of Andersson et al.^14^ predicted an increase that ranges from 5.1 to 19.2 mmHg at ROC of 1,000 ft/min. This discrepancy may be due to the underlying assumption of the model of Andersson et al.^14^, which assumed the pressure inside the ICA to equilibrate with the surrounding cabin pressure^14^. In other words, the model assumes the ICA to expand until its pressure is equivalent to that of the cabin. This assumption is not entirely correct, as the stiffness of the agarose gel (brain) and the resistance from the water balloon (CSF contained inside the ventricle system) are likely to restrict the amount of expansion of the ICA.

Based on the analysis carried out in “Effects of initial ICP” section, it was determined that an initial ICP of 16.45 mmHg is required for ICP_cruise_ to breach the 20 mmHg safe limit, which is applicable for ICA volume of 20 ml and ROC of 960 ft/min. Assuming that the independence between ΔICP and initial ICP is valid also for ICA volumes of 10 and 15 ml, then the initial ICP required for ICP_cruise_ to breach 20 mmHg would have been larger than 16.45 mmHg since the ΔICP for these volumes were smaller than that for 20 ml (see Table 1).

A similar argument can be made for the case when ROC = 1,600 ft/min. From Table 1, the ΔICP obtained for ICA volume of 20 ml and ROC = 1,600 ft/min is 5 mmHg. Hence, the required initial ICP to breach the 20 mmHg safe limit would be 15 mmHg, while smaller ICA volumes would elevate the required initial ICP. For ROC that are smaller than 960 ft/min, the initial ICP that is safe for air travel is expected to be larger than 16.45 mmHg based on the finding that ΔICP decreases with decreasing ROC. Hence, one may deduce from this analysis that the conservative threshold of initial ICP for a pneumocephalus patient to safely embark on air travel is 15 mmHg or lower for ICA volume of 20 ml or smaller regardless of the ROC.

The formation of cracks across the surface of agarose gel reported in “Effects of ICA volume ≥ 30 ml” section suggests that it may not be safe for pneumocephalus patients with ICA volumes of 30 ml or larger to embark on air travel, as this may lead to complications due to elevated ICP and cerebral herniation. Large expansion of ICA may also lead to more severe neurological disorders such as epilepsy^29^ and focal neurological deficits, depending on the location of the ICA within the brain^30,31^. Although the experiments performed in “Effects of ICA volume ≤ 20 ml and ROC” section were limited only to 15 mmHg, the 30 ml threshold for unsafe travel applies also to other values of initial ICP. This is due to ΔICP being dependent only on the degree of ICA expansion (Boyle’s law) and not the initial ICP. On the other hand, air travel is likely to be safe for patients with ICA volumes of 20 ml or smaller if the initial ICP is 15 mmHg or smaller.

It is noteworthy that the aforementioned recommendation represents only a conservative estimate, i.e. a cautious representation of the actual threshold due to the present experimental model not accounting for the more complex CSF circulation system. The actual threshold may be much higher as the near-spherical shape of the air balloon resulted in a low surface area of contact between the balloon and the gel, which allowed the gel to crack more easily. In reality, the shape of ICA varies, which may induce different physical responses within the brain. Realistically, adverse neurological effects may occur even at ICP that is lower than 25 mmHg. For example, cerebral herniation may occur at ICP as low as 20 mmHg under certain circumstances, such as in the presence of a temporal mass lesion^18^. Therefore, the aforementioned recommended threshold of ICA volume and initial ICP for safe air travel is based solely on the effects of ICA expansion and may not be applicable if the patient has other medical complications.

During the experiments, a rapid drop and a rapid rise in ICP were recorded during the initial ascending and descending stages, respectively. This was determined to be a combination of the partial exposure of the gel surface to the ambient air and the use of the highly-sensitive digital pressure gauge. As the air is pumped out of the pressure chamber, the weight (mass) of the air that is acting on the gel through the area of partial exposure decreases. This causes the initial drop in the ICP reading that was picked up by the highly-sensitive pressure gauge. Since air is contained inside the latex balloon, the expansion must overcome the resistance from the balloon elasticity before any force can be exerted onto the water balloon to increase its ICP. A similar explanation applies for the rise in ICP during the initial stages of descending.

Some limitations to the present study must be highlighted in order for them to be addressed in future studies. Firstly, the intracranial system adopted in the present study was a simplified version that consisted of only the brain, the ICA and the CSF. In reality, the CSF is contained inside the ventricular system that consists of four separate components. Moreover, the CSF can also be found in the subarachnoid space that surrounds the brain tissue. Secondly, the present model has not included the compensatory mechanisms, i.e. the shift of venous blood and CSF out of the intracranial compartment that limits the rise in ICP during ICA expansion. It is difficult to incorporate these anatomical details into the existing experimental setup; however, it is acknowledged that these differences may also influence how the ICP responds to the expansion of ICA and hence, they are worthy of future considerations. In spite of this, the present experimental model may still explain scenarios that represent an actual clinical setting. It is well-known in the medical community that much of the CSF is removed following craniotomy. This CSF is not expected to be fully replenished in the first few weeks of surgery, as indicated by CT scans of the head that continue to show intracranial air and slumping of the brain. The intracranial environment during this period is very similar to the experimental model set up in the present study.

Thirdly, the use of a latex balloon to contain the air volume may result in some degree of error in its expansion. This is due to the resistance provided by the elastic properties of the latex that the expansion forces must overcome before any actual expansion can occur. Given this circumstance, the actual ICP reading may be larger than what was presented in “Results” section. Lastly, the shapes of the cranium and the brain were assumed to be inconsequential to the expansion of ICA and the elevation in ICP. An anatomically more realistic model that is achievable through 3D printing technology may be considered in future studies. This should allow for a more realistic placement of the ICA and a more accurate representation of the ICA expansion.

## Conclusions

The postulated effect of ICA expansion and the subsequent increase in ICP in a pneumocephalus patient embarking on air travel has been successfully demonstrated and quantified in a simulated laboratory setting, which to the best of the authors' knowledge, is the first of its kind.

Results obtained from the present study showed that the rise in ICP increases with both ICA volume and ROC. However, the influence of initial ICP on the rise in ICP was insignificant. Based on the quantitative and qualitative examinations of the experimental findings, air travel is not recommended for pneumocephalus patients with ICA volume of 30 ml or larger in order to avoid complications associated with severe ICP elevation and cerebral herniation. On the other hand, an initial ICP of 15 mmHg and ICA volume of 20 ml were recommended as conservative thresholds for safe air travel for ROC less than or equal to 1,600 ft/min.

The successful demonstration and quantification of the ICA expansion and its associated increase in ICP in the laboratory is crucial as it allows future studies on air travel safety among pneumocephalus patients to be carried out in a cost-effective way and in a controlled environment. This study provides laboratory evidence on which clinical decisions and airline policies can be made on whether a patient with pneumocephalus can safely fly.
